# Medical Statistics

**Published:** 1837-01

**Authors:** 


### MEDICAL STATISTICS.

### *Statistics of Calculus in Austria.*

THE following tabular views are extracted from VON WATTMANN's recent Treatise on Lithotrity and Lithotomy, (*Ueber die Steinzerbohrung und ihr Verhältniss zum Blasenschnitte.* Wien, 1835. 8vo.) and are founded on official documents supplied by Professor Raimann, of Vienna.

I. *Calculous Cases in the Austrian Dominions, from* 1820 *to* 1830.

| Provinces. | Whole Population. | No. of Calculous Cases. |
|---|---|---|
| Vienna and Lower Austria, including the military, | 1,206,520 | 49 |
| On the Ems and Salzburg | 835,043 | 18 |
| Galitzia | 4,316,086 | 19 |
| Moravia | 2,046,787 | 39 |
| Bohemia | 3,582,150 | 106 |
| The Tyrol | 780,399 | 11 |
| Styria | 854,720 | 10 |
| Illyria and Maritime States | 1,154,885 | 31 |
| Venice and the Eight Provinces | 2,032,339 | 278 |
| Milan and Lombardy | 2,400,000 | 794 |
| Dalmatia | 383,600 | 49 |
| Total | 19,592,529 | 1,449 |

Proportion, one case in 13,531 of the population.

II. *Cases of Lithotomy in the Surgical Clinic of Vienna, during thirty-five years.*

| Age of the Patients. | No. operated on. | Deaths. |
|---|---|---|
| From 1 year to 10 | 71 | 7 |
| — 11 — 20 | 42 | 3 |
| — 21 — 30 | 30 | 9 |
| — 31 — 40 | 12 | 3 |
| — 41 — 50 | 6 | 3 |
| — 51 — 60 | 11 | 6 |
| — 61 — 68 | 8 | 3 |
| Total | 180 | 34 |

All the patients admitted, except four, were operated on. The above table gives the following results :

| | |
|---|---|
| Proportion of deaths to the whole operations, | 1 in $5\frac{1}{4}$ |
| in children . | 1 ... $11\frac{3}{10}$ |
| between the ages of 20 and 30, | 1 ... $3\frac{8}{11}$ |
| — 50 and 60, | 1 ... $2\frac{1}{11}$. |

Of the thirty-four deaths, the following are given as the causes:—Gangrene of the bladder, 6; Phthisis, or hectic fever, 5; Debility, 6; Exhaustion from Suppuration, 3; Nervous shock, 3; Typhus, 6; Convulsions, 1; Suppuration of the Kidneys, 1; Cholera, 1.

III. *General Chemical Constitution of the Calculi in the Vienna Collections.*

| Nature of the Calculus. | In the Pathological Museum. | In the University Museum. | In the Joseph Museum. | In V. Kern's Collection. | In Watmann's Collection | Total |
|---|---|---|---|---|---|---|
| Phosphatic . | 32 | 26 | 27 | 28 | 26 | 139 |
| Oxalic acid, with phosphatic envelope | 3 | 16 | 5 | 8 | 8 | 40 |
| Oxalate of lime . | 4 | 12 | 3 | 1 | 7 | 27 |
| Oxalic acid, with uric acid envelope. | 9 | 3 | ... | 3 | 5 | 20 |
| Uric acid . | 11 | 9 | 10 | 10 | 11 | 51 |
| Number of patients and calculi . | 59 | 66 | 45 | 50 | 57 | 277 |

*Schmidt's Jahrbücher der Gesammten Medicin*, B. x. H. 3, No. 5, 1836.

---

### *Medico-Statistical Report of the Deaf and Dumb in the Dutchy of Brunswick, in the year* 1835. By Dr. MANSFELD.

(1.) THE whole population of the dutchy, 253,232; the total number of deaf and dumb, 125; consequently, the proportion is 1 in 2,026. (2.) Of the 125, 60 are males, and 65 females. (3.) In Prussia, the proportion of deaf and dumb to the population is 1 in 1,426. (4.) Nearly the fourth part of the whole number had one or two brothers or sisters similarly affected. (5.) For the most part, these persons belong to the middle and lower classes; their parents being generally poor. (6.) The health of these persons is in general good; those residing in the vicinity of the Harz are said to be scrofulous, and five of the whole number are idiotic. In two cases only could the deaf-dumbness be traced to distinct causes; viz. one as the consequence of fright, the other of miliary fever. (7.) Almost all the deaf-dumb in the dutchy have the benefit of education; a circumstance very creditable to the country, and which the author, with a just pride, contrasts with the great neglect of the same class of persons in Austria, in which vast empire it appears that, out of 20,639 individuals labouring under this infirmity, only 400 are placed in houses of instruction, (ten in number;) all the rest being left without assistance. When it is considered that, of this number, the fifth part at least are susceptible of instruction, there is evidently here a great neglect of the duties of humanity. (8.) With the exception of those who are yet too young to work, or who are mentally incapable of gaining their livelihood, or are supported by relatives, (57 in all,) or who are in the course of instruction, (number not mentioned,) all the others are gaining their own livelihood as artisans and labourers. (9.) Dr. Mansfeld calls the particular attention of teachers to the fact, that, in many cases, the inability to acquire the sound of particular letters or words depends on physical defects of the organs of speech, and not on mental incapacity. In proof of this he instances nine cases among the children at this time in the Brunswick Institution, who labour under some defect of this kind. The defects mentioned are the following:—Imperfect uvula; thick tongue, without frænum; large tongue, long and irregular uvula; tongue deficient in muscularity; tubular palate, imperfect uvula; enlarged tonsils; flat and irregular palate; general defective size of mouth, large tonsils; imperfectly developed larynx.

*Hannoversche Annalen*, B. i. H. 4. *October*, 1836.

